# Resilience, COVID-19-related stress, anxiety and depression during the pandemic in a large population enriched for healthcare providers

**DOI:** 10.1038/s41398-020-00982-4

**Published:** 2020-08-20

**Authors:** Ran Barzilay, Tyler M. Moore, David M. Greenberg, Grace E. DiDomenico, Lily A. Brown, Lauren K. White, Ruben C. Gur, Raquel E. Gur

**Affiliations:** 1 University of Pennsylvania Perelman School of Medicine, Department of Psychiatry, Philadelphia, PA USA; 2 Lifespan Brain Institute of the Children’s Hospital of Philadelphia and Penn Medicine, Philadelphia, PA USA; 3 Children’s Hospital of Philadelphia Department of Child Adolescent Psychiatry and Behavioral Sciences, Philadelphia, PA USA; 4 Bar Ilan University, Ramat Gan, Israel

**Keywords:** Scientific community, Depression, Scientific community, Depression

## Abstract

COVID-19 pandemic is a global calamity posing an unprecedented opportunity to study resilience. We developed a brief resilience survey probing self-reliance, emotion-regulation, interpersonal-relationship patterns and neighborhood-environment, and applied it online during the acute COVID-19 outbreak (April 6–15, 2020), on a crowdsourcing research website (www.covid19resilience.org) advertised through social media. We evaluated level of stress (worries) regarding COVID-19: (1) contracting, (2) dying from, (3) currently having, (4) family member contracting, (5) unknowingly infecting others with (6) experiencing significant financial burden following. Anxiety (GAD7) and depression (PHQ2) were measured. Totally, 3042 participants (*n* = 1964 females, age range 18–79, mean age = 39) completed the resilience and COVID-19-related stress survey and 1350 of them (mean age = 41, SD = 13; *n* = 997 females) completed GAD7 and PHQ2. Participants significantly endorsed more distress about family contracting COVID-19 (48.5%) and unknowingly infecting others (36%), than getting COVID-19 themselves (19.9%), *p* < 0.0005 covarying for demographics and proxy COVID-19 exposures like getting tested and knowing infected individuals. Patterns of COVID-19 related worries, rates of anxiety (GAD7 > 10, 22.2%) and depression (PHQ2 > 2, 16.1%) did not differ between healthcare providers and non-healthcare providers. Higher resilience scores were associated with lower COVID-19 related worries (main effect *F*_1,3054_ = 134.9; *p* < 0.00001, covarying for confounders). Increase in 1 SD on resilience score was associated with reduced rate of anxiety (65%) and depression (69%), across healthcare and non-healthcare professionals. Findings provide empirical evidence on mental health associated with COVID-19 outbreak in a large convenience sample, setting a stage for longitudinal studies evaluating mental health trajectories following COVID-19 pandemic.

## Introduction

The COVID-19 pandemic is impacting humankind in unprecedented and monumental ways and data is needed to plan for next steps following the acute outbreak^1^. In addition to physical health, coping with the pandemic requires mental resilience. Tools have been established to estimate resilience, broadly conceptualized as healthy and adaptive functioning in the aftermath of adversity^2^. Measuring resilience can (1) allow better planning of resource allocation and (2) inform interventions for individuals and communities to overcome the acute pandemic effects^3^ expected to impact mental health^4^. Healthcare providers are on the frontlines of the pandemic response and already show deleterious mental health consequences^5^. Hence, there is an urgent need to gauge the role of resilience specifically in this population^6^.

The internet has transformed our ability to collect large-scale data through crowdsourcing, with rapid outreach to large samples complying with social distancing^7^. We previously developed and applied a tool to measure resilience using self-report items^8^. Here, we applied an interactive online platform to measure resilience in a population enriched for healthcare providers. We hypothesized that (1) COVID-19 related stress (estimated by subjective worries) will be associated with generalized anxiety and depression; (2) higher resilience scores would correlate with less worries, generalized anxiety and depression; (3) healthcare providers will report higher levels of COVID-19 related concerns, anxiety and depression. We also explored differences in COVID-19 related stress and resilience between participants from US and Israel.

## Subjects and methods

### Participants and procedures

On April 6th 2020, we launched a website (https://www.covid19resilience.org/) that included an interactive 21-item resilience survey and assessment of COVID-19-related stress (worries) regarding: (1) getting (contracting), (2) dying from, (3) currently having, (4) family member getting, (5) unknowingly infecting others, and (6) experiencing significant financial burden following COVID-19. Participants were asked to rate how much they worried on a 5-item scale (0—not at all; 1—a little; 2—a moderate amount; 3—a lot; 4—a great deal). At the end of the survey, participants received feedback on their resilience scores with personalized recommendations regarding stress management. The feedback was also meant to incentivize participants to complete the survey carefully. Next, participants were offered to take a second survey on their anxiety (generalized anxiety disorder 7 questionnaire (GAD7))^9^ and depression (patient health questionnaire 2 (PHQ2))^10^. The study was advertised through, (1) the researchers’ social networks, including emails to colleagues around the world; (2) social media; (3) the University of Pennsylvania and Children’s Hospital of Philadelphia internal notifications; and (4) organizational mailing lists. In addition to English, the survey was available in Hebrew after a two-way reverse translation and consensus by three bilingual English-Hebrew speakers. The results presented here are based on data collected from April 6 to 15, 2020. Participation required responders to provide online consent. The study was approved by the Institutional Review Board of the University of Pennsylvania.

### Resilience survey

The survey was based on questions associated with resilience that were recently compiled into a single battery^8^. The items included were identified following administration of 212 items to >250 participants. The 212 items were reduced, using factor analysis followed by computerized adaptive test simulation, to a 47-item battery comprising seven factors^8^. For the sake of brevity and scalability of the online survey, we use five of the seven factors, resulting in a 21-item abbreviated version: self-reliance (3 items)^11^; emotion regulation (5 items)^12^; positive (4 items) and negative (5 items)^13^ relationship characteristics; and neighborhood characteristics (4 items)^14,15^. Resilience items included in the survey and their corresponding scores are described in Supplementary Table 1. To create a resilience score, we summed the score on all 21 items after coding them such that a higher score always indicates higher resilience.

### Data analysis

For COVID-19 related worries, all main and interaction effects were investigated using mixed models to account for within-person variance across items. The mixed model treated the 6 items of COVID-19 worry/stress (evaluated at the same time in this cross-sectional study) as repeated measures within individual. The key dependent variable was item response (5-point scale indicating level of COVID-19-related worry), and we addressed the following questions: (1) Are certain types of worry more common than others? (2) Are there sex differences or age-related effects on the type of worry? (3) Is resilience associated with lower worry? (4) Does the effect of resilience depend on the type of worry? (5) Do the pattern of effects differ in healthcare providers? All models included the following potential confounders: age, gender, race (white/non-white), education, income, occupation (healthcare, engineering/computers and other), marital status (married, single or other), country of residence (US, Israel or other), number of people in household, date at which the survey was taken (days and (days squared) since study launch), and exposures related directly to COVID-19 including getting tested for COVID-19, knowing someone who tested positive for COVID-19 and knowing someone who died from COVID-19. All analyses were performed using the *lmerTest*^16^ package in R.

To evaluate the association of COVID-19 related stress with anxiety and depression, we used regression models with COVID-19-related worries (standardized *z*-score of the sum all 6 worry questions) as the independent variable. The key dependent variable was either a dichotomized measure of meeting screening levels of GAD and depression (binary logistic regression); or continuous GAD7 and PHQ2 score (linear regression). Based on reports of overall higher scoring in anxiety^17^ and depression^18^ in web-based compared to paper and pencil surveys, we chose more conservative cutoffs to capture moderate and above anxiety or depression. GAD7 score > 10 was considered a case of probable generalized anxiety^19^, PHQ2 score > 2 was considered a case of probable depression^20^. Models covaried for age, gender, race, education, income, occupation, marital status, country of residence, number of people in household, and date at which the survey was taken.

To evaluate the mitigating effect of resilience on generalized anxiety and depression, resilience score was considered as the independent variable, with continuous GAD7 or PHQ2 score (using linear regression) or dichotomous GAD/depression (using binary logistic model) as the dependent variables. All models included multiple co-variates as described above. Effects of gender, age, and being a healthcare provider were tested in separate models including interaction terms of all of the above with resilience overall score.

In an exploratory analysis we compared participants from the US and Israel using regression models with US/Israel as a binary independent variable and COVID-19 worries/stress, resilience, anxiety, and depression as the dependent variables. Models included all covariates listed above.

All regression analyses were conducted in SPSS version 26 (IBM).

## Results

We obtained data from 3042 participants 10 days into the study. The majority of participants were female (*n* = 1964, 64.6%), with a wide age range from 18 to 79 years (M = 38.9, SD = 11.9). Due to the method of advertisement through the researchers’ social networks and University/Hospital announcements, the sample was enriched for academics (54% Master/Doctorate degree) and healthcare providers (20.5% of sample were physicians (*n* = 312)/nurses (*n* = 106)/other healthcare with direct patient care (*n* = 208)). Demographics are shown in Table 1.

**Table 1:** Sample demographics^a^ (*N* = 3042).

	*n*	%
Age bins		
Under 30	719	23.6
30 s	1043	34.3
40 s	741	24.4
50 s	335	11
60 s	157	5.2
Over 70	47	1.5
Other demographics		
Gender, female	1964	64.6
Gender, male	1059	34.8
Race, white	2577	84.7
Relationship		
Married/living with partner	2148	70.6
Single	557	18.3
Occupation		
Healthcare	625	20.5
Engineering, computers, finance	506	16.7
Research	344	11.3
Legal, government, administration	271	8.9
Student	269	8.8
Teaching	200	6.6
Education		
Bachelor or lower	1384	45.5
Master degree	1035	34
Doctoral degree	615	20.2
Income (annual per household)^b^		
Under $50,000	798	26.2
$50,000 to $99,999	705	23.2
$100,000 and above	1296	42.6
Country of residence		
US	1607	52.8
Israel	1197	39.3
Other^c^	238	7.8
COVID-19 exposures		
Tested negative for COVID-19	132	4.3
Tested positive for COVID-19	12	0.4
Know personally person with COVID-19	1276	41.9
Know personally person who died from COVID-19	191	6.3

^a^Missing demographic data for participants answering “I don’t know/I’d rather not say” was lower than 1.8% for all variables except income.

^b^Missing data for income = 8.8%.

^c^Other countries included UK (*n* = 50), Canada (*n* = 30), Brazil (*n* = 17), Germany (*n* = 15), Ireland (*n* = 11), and 42 other countries with less than 10 participants.

### COVID-19-related worries

**Fig. 1:**
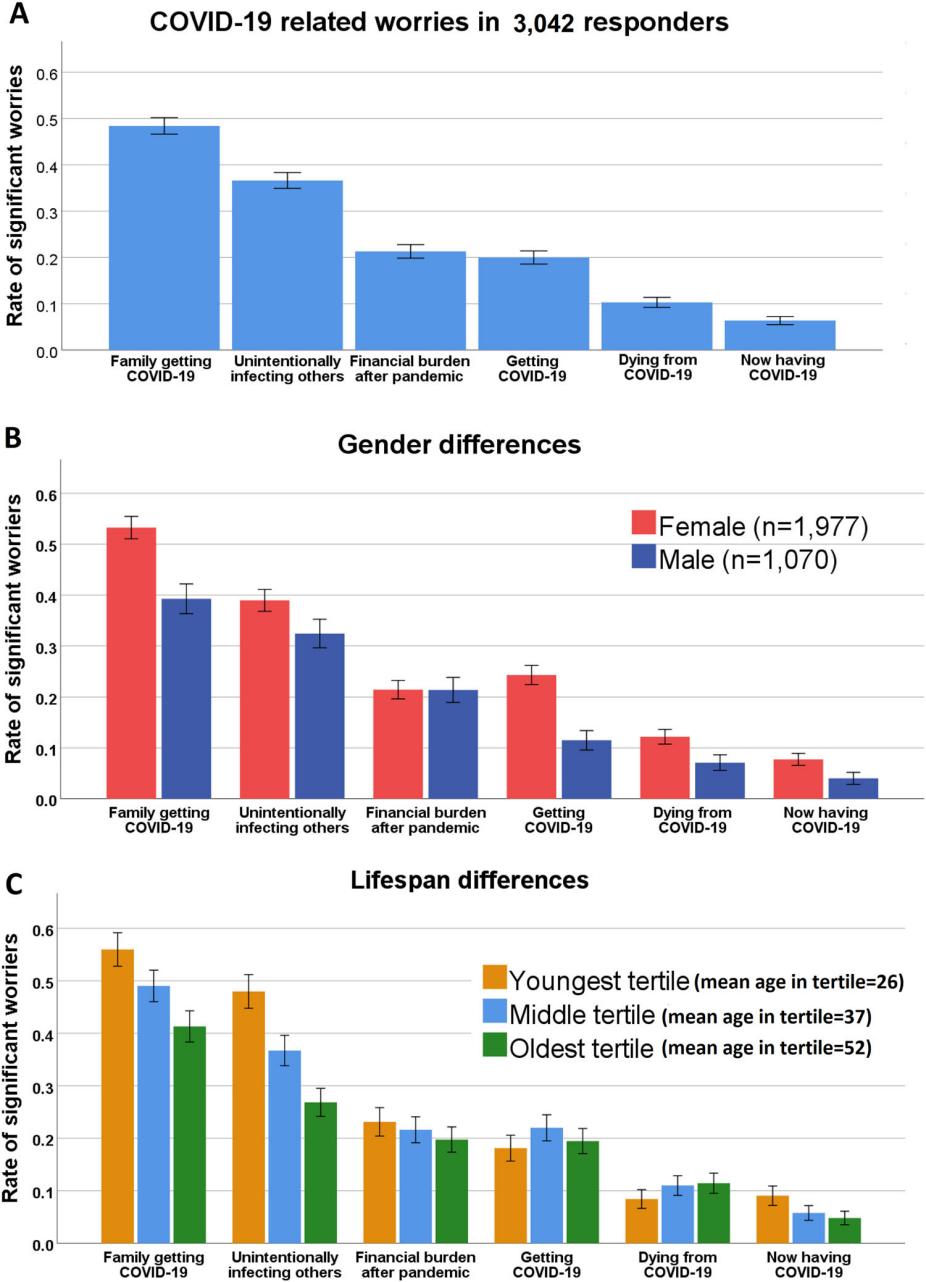
COVID-19-related stress in study participants (A) with gender (B) and age (C) comparison. **a** Patterns of COVID-19-related worry in the entire sample; **b** gender differences; **c** age differences. *y*-axis represents the rate of responders endorsing significant worry (a lot/a great deal, items 4/5 on a 5 option Likert scale). Error bars represent 95% confidence intervals.

Participants were significantly more worried about a family member contracting COVID-19 or about unknowingly infecting others than about getting COVID-19 themselves (Fig. 1a, item main effect *F*_5,15205_ = 1536.0, *p* < 0.00001, model included age, gender, education, income, marital status, number of people in household and country of residence). Participants worried to a similar extent about financial burden following COVID-19 as about getting COVID-19; worried less about dying from COVID-19; and worried least about currently having COVID-19. Females had overall higher COVID-19-related worries than males, except for the financial burden, where they were comparable to males (Fig. 1b, item-by-sex interaction *F*_5,15200_ = 25.9; *p* < 0.00001). The pattern of worrying more about others compared to self was consistent throughout the lifespan. Older participants worried more about themselves than their younger counterparts, but still worried more about others (Fig. 1c, item-by-age interaction *F*_5,15200_ = 71.6; *p* < 0.00001).

### Anxiety and depression

**Fig. 2:**
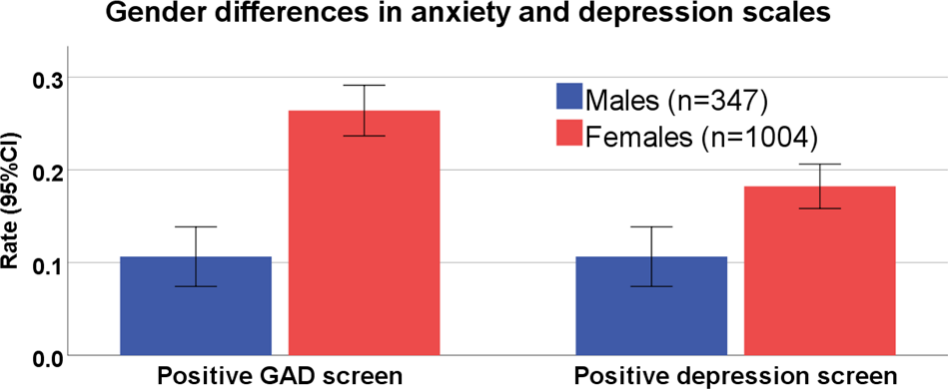
Gender differences in anxiety and depression. A positive GAD screen was considered for in GAD7 score > 10. Positive depression screen was considered for PHQ2 score > 2. GAD generalized anxiety disorder.

We evaluated generalized anxiety and depression in a subsample of *n* = 1350 who completed the GAD7 and PHQ2 questionnaire (Fig. 2). This subsample did not differ from the participants who did not complete GAD7/PHQ2 scales (*n* = 1692) in terms COVID-19-related worries (worry sum score, *t* test, *p* = 0.387) or composite resilience score (*t*-test *p* = 0.932). Female gender was associated with higher scores on GAD7 (standardized beta = 0.143, *p* < 0.001) and PHQ2 (standardized beta = 0.069, *p* = 0.03), and with higher rates of meeting threshold screening for GAD (OR = 1.93, 95% CI: 1.24–2.99, *p* = 0.004), but not with meeting threshold depression (OR = 1.47, 95% CI: 0.93–2.32, *p* = 0.103). Older age was associated with lower likelihood of meeting threshold anxiety (OR = 0.97, 95% CI: 0.95–0.98, *p* < 0.00001) but not for depression (*p* = 0.17). Models included race, education, income, occupation, country of residence and number of people in household as co-variates.

### Association among COVID-19-related worries, generalized anxiety, and depression

Higher endorsement of COVID-19-related worries was strongly associated with meeting threshold screening for generalized anxiety (GAD7 score > 10, *n* = 300, 22.2%) and depression (PHQ2 score > 2, *n* = 217, 16.1%), such that for every 1 SD increase in the standardized composite score of COVID-19 worries, there was more than 2-fold increased probability of generalized anxiety (binary logistic regression OR = 2.23 95% CI: 1.88–2.65, *p* < 0.001; linear regression standardized beta = 0.396, *t* = 15.571, *p* < 0.001) and 67% increased probability of depression (binary logistic regression OR = 1.67 95% CI: 1.41–1.98, *p* < 0.001; linear regression standardized beta = 0.212, *t* = 7.266, *p* < 0.001). There was no difference in the strength of association between different types of COVID-19 related worries (to self, others or financial burden) and anxiety or depression (Supplementary Table 2). Models covaried for age, gender, education, occupation, income, marital status, number of people in household, country of residence, and date taking the survey.

### Association of resilience score with COVID-19-related worries, generalized anxiety, and depression

**Fig. 3:**
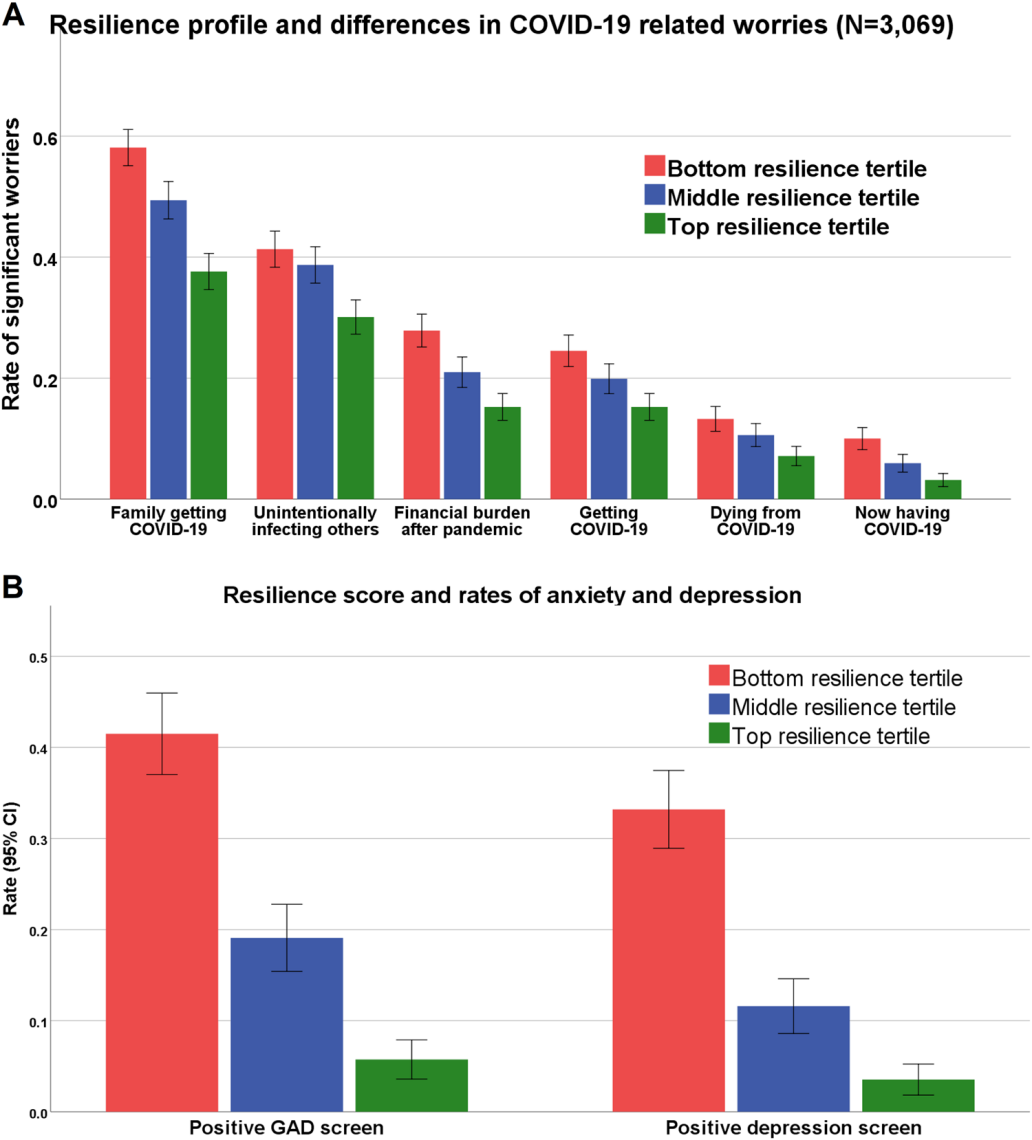
Resilience profile association with (A) COVID-19-related worries and with (B) anxiety and depression rates. **a** *Y*-axis represents the rate of responders endorsing significant worry (a lot/a great deal, items 4/5 on a 5 option Likert scale). Error bars represent 95% confidence intervals. **b** A positive-GAD screen was considered for in GAD7 score > 10. Positive depression screen was considered for PHQ2 score > 2. GAD generalized anxiety disorder.

The composite resilience score derived from the 21-item survey buffered all the COVID-19-related worries, such that participants with higher resilience scores worried significantly less than low scoring individuals about COVID-19 (Fig. 3a, main effect *F*_1,3023_ = 134.9; *p* < 0.00001). Furthermore, higher resilience scores were associated with lower generalized anxiety (total GAD7 score, linear regression standardized beta −0.418, *t* = −16.44, *p* < 0.001) and depression (total PHQ2 score, linear regression standardized beta −0.451, *t* = −16.72, *p* < 0.001). The effect was such that for every 1 SD increase in the resilience score there was a 64.9% decrease in the possibility of positive-GAD screen (binary logistic regression OR = 0.351, 95% CI: 0.29–0.424, *p* < 0.0001, Fig. 3b) and a 69.3% decrease in the possibility of positive depression screen (OR = 0.31, 95% CI: 0.252–0.383, *p* < 0.0001, Fig. 3b).

The inverse association of high resilience score with meeting screening threshold of anxiety and depression was consistent across genders (resilience by gender interaction nonsignificant). Overall, the mitigating association of higher resilience with lower probability of anxiety was stronger in older age (resilience score by age interaction Wald = 6.955, *p* = 0.008 for GAD), with trend level significance for depression (resilience score by age interaction Wald = 3.378, *p* = 0.066).

### Comparison between US and Israel participants

The majority of our study sample were from the US (*n* = 1607) or Israel (*n* = 1197). We conducted exploratory comparisons between samples from the two countries. Participants from the two countries differed on demographics (Supplementary Table 3). Except for age that was similar in both countries, the US sample included more females (84 vs. 39%), more healthcare providers (29 vs. 11%) with higher education and higher income compared to Israel.

Multivariate comparison that co-varied for multiple confounders revealed that participants from the US were overall more worried/stressed about COVID-19 (Table 2). US participants were specifically more stressed about self (contracting COVD-19, dying from COVID-19 and currently having COVID-19) compared to Israel participants, with no differences in worries about others (family getting COVID-19/infecting others) or about financial burden due to COVID-19 (Table 2). Israel participants scored higher overall on the resilience scale (standardized beta = 0.163, *t* = 5.694, *p* < 0.001). US participants were more likely to meet screening criteria for GAD (OR = 4.9, 95% CI: 2.6–9.4, *p* < 0.001) and for depression (OR = 2.2, 95% CI: 1.2–4, *p* < 0.001).

**Table 2:** COVID-19 worries, anxiety and depression among US participants compared to Israel participants.

Worries/stress	Standardized beta^a^	*P* value
Overall COVID-19 worries/stress	0.107	<0.001
Contracting COVID-19	0.175	<0.001
Dying from COVID-19	0.115	<0.001
Currently having COVID-19	0.136	<0.001
Family contracting COVID-19	0.058	0.071
Infecting others with COVID-19	0.022	0.498
Financial burden *d*/*t* COVID-19	−0.032	0.266

Anxiety/depression screening	Odds ratio^b^ (95% CI)	*P* value
GAD7-positive screen	4.94 (2.6–9.39)	<0.001
PHQ2-positive screen	2.15 (1.16–3.98)	0.015

^a^Values derived from linear regression models with US/Israel (binary variable) as the independent variable and the worry/stress item as the dependent variable. Models included the following co-variates: age, gender, race, marital status, occupation, education, number of people in household, getting tested for COVID, knowing someone who tested positive from COVID or who died from COVID and date of survey completion.

^b^Values derived from binary regression models with US/Israel (binary variable) as the independent variable and positive GAD screen (>10) or positive-PHQ screen (>2) as the dependent variable. Models co-varied for age, gender, race, marital status, occupation, education, number of people in household, and date of survey completion.

### Sensitivity analyses in healthcare providers

Due to the high percentage of healthcare providers (physicians, nurses, and other direct patient care, *n* = 625) in this sample, we repeated the above analyses including interactions with healthcare profession status. In COVID-19-related worries, the only difference was that healthcare providers worried more than non-healthcare providers about contracting COVID-19 (*t*_15340_ = 3.9, *p* < 0.0005) and less than non-healthcare providers about finances after COVID-19 (*t*_15340_ = −6.9, *p* < 0.00001, Supplementary Fig. 1). We did not detect higher anxiety and depression in healthcare providers compared to non-healthcare providers (Supplementary Fig. 2).

Higher resilience scores were associated with less COVID-19-related worries similarly across healthcare providers and non-health care professionals (main effect *F*_1,3053_ = 102.0, *p* < 0.00001; resilience by healthcare providers interaction nonsignificant, Supplementary Fig. 3). Similarly, higher resilience scores were associated with lower likelihood of meeting GAD or depression screening threshold across professions (resilience by healthcare providers interaction nonsignificant, Supplementary Fig. 4).

## Discussion

The rapid spread of COVID-19 creates a unique opportunity to evaluate resilience in the face of a single global adversity. Here, we captured a unique snapshot for over 3000 people who were in stressful conditions during the acute pandemic outbreak (>92% of our sample are from US or Israel that were in lockdown during the study period). Participants reported significantly more subjective worries (stress) about others (~50% worried about family member getting COVID-19) than about getting COVID-19 themselves (~20%). This pattern was consistent across genders, throughout the lifespan and was overall similar in healthcare providers compared to non-healthcare providers. This finding is consistent with work reporting increased prosocial behavior under stress^21^, and may be related to “tend-and-befriend”, where in response to threat humans tend to protect their close ones (tending) and seek out their social group for mutual defense (befriending)^22^. This finding might be interpreted as a form of altruism during acute stress of the pandemic outbreak. Notably, altruistic behavior described in acute situations throughout history was previously linked to mechanisms of resilience for overcoming adversity^23^.

The COVID-19-related worries were associated with substantial levels of anxiety (22%) and depression (16%) in the subsample that completed the GAD7 and PHQ2 questionnaires (*n* = 1350). These rates are higher than previously reported point prevalence rates^24,25^. Several explanations may account for the higher reported levels of anxiety and depression. First, it was previously described that people report more symptoms in web-based surveys^17,18^. Second, our sample was enriched for women, who are known to report more anxiety and depression. In that sense the expected gender differences we observed support the validity of our data^26^. Lastly, it is possible that during the acute phase of the pandemic when the data was collected, and in light of the high level of stress and worries related to COVID-19, there are higher levels of anxiety and depression in the population as reflected in our convenience sample. The rates we report here are also higher than the rates reported in healthcare providers during the acute COVID-19 outbreak in China^5^. This effect might be explained by cultural differences or difference in sampling, as we used an online survey and the Chinese study sampled through hospitals.

Supporting our hypothesis, the brief online resilience survey inversely correlated with COVID-19 worries, generalized anxiety and depression symptoms. The survey may tap into traits and factors that allow a buffering against COVID-19 related stressors. This “buffering effect” was evident in both genders, throughout the lifespan, and in a similar manner in healthcare and non-healthcare providers. Notably, while there is need for more longitudinal data on resilience^27^, scarce longitudinal data suggests that baseline resilience mitigates developing anxiety and depression following adversity^28^. Therefore, the framework described in this study can be used in longitudinal studies that evaluate trajectories of mental health conditions and needs following the pandemic outbreak.

This study’s main strength is the large sample and the unique timing of data collection, in which vast majority of the sample (>90% are from the US or Israel) were in lockdown, with closures of school and nonessential businesses. These unusual life circumstances are likely to have major impact on mental health^29^, and thus provide an opportunity to study resilience in the face of a global stressor. Despite the global nature of the stressor, we found significant differences between participants from Israel and the US, the latter reporting more stress, anxiety and depression. The reasons for these difference require further investigation, but it should be noted that the two countries greatly differ is their size in terms of geographic area, total population, GDP, in addition to differences in other social, cultural, political, economic and health system characteristics. Our findings might imply that local factors may contribute to the levels of stress, resilience and mental health at times of global pandemic. Specifically, it is possible that residents of countries more accustomed to dealing with collective stressors (such as Israel, due to frequent war-related events), can recruit more resilience factors during an acute stressor such as a pandemic outbreak. Future studies are needed to evaluate whether these differences between countries are maintained longitudinally, beyond the acute stress of the outbreak, as the stress is likely to shift from the medical consequences of COVID-19 to the economic impact.

Several study limitations should be considered. These include the biased sampling to a more educated, professional population that is enriched for healthcare providers and academics. More data is required from other sociodemographic backgrounds that appear to be more vulnerable^30^. There are also the inherent limitations of data collection through crowdsourcing (i.e., how generalizable are people who complete online surveys)^31^. However, we did not pay participants, but rather provided feedback based on their responses, which mitigates the concern that a participant deliberately answered inaccurately, as a main incentive to take the survey was to receive personalized feedback. In addition, we used brief screening measures for anxiety and depression. As for the healthcare providers’ data, we did not collect data regarding exposure to COVID-19 patients, militating our ability to link this exposure form to the “pandemic response frontline” to the measures studied here. Lastly, the cross-sectional design does not allow causal inferences, which can be addressed in future longitudinal studies.

To conclude, we present data collected from a large convenience sample in the acute phase of the COVID-19 pandemic, when the majority of the sample was bound to a “lockdown” with severe social distancing. We report two main findings: (1) People are worried more about others than about self when reporting COVID-19 concerns; (2) Resilience helps reduce worries as well as anxiety and depression. Longitudinal studies are needed to address whether resilience scores are consistent and whether they can predict trajectories of mental and general health as humanity moves toward the post-COVID-19 pandemic era.

## Supplementary information

Supplemental Material
