# Supplementary material for: Resilience, COVID-19-related stress, anxiety and depression during the pandemic in a large population enriched for healthcare providers
Source: Transl Psychiatry. 2020 Aug 20;10:291. doi: 10.1038/s41398-020-00982-4 (PMC7439246; doi:10.1038/s41398-020-00982-4)
Supplement: Supplementary file 1 — Supplemental Material [file 41398_2020_982_MOESM1_ESM.docx]

**Supplementary Material-**

**Supplementary eFigures 1-4:** Comparing Healthcare providers (physicians (n=312), nurses (n=106), other healthcare with direct patient care (n=208)) to non-healthcare providers.

**Supplementary eFigure 1-** COVID 19 related stress comparing healthcare providers to non-healthcare providers.


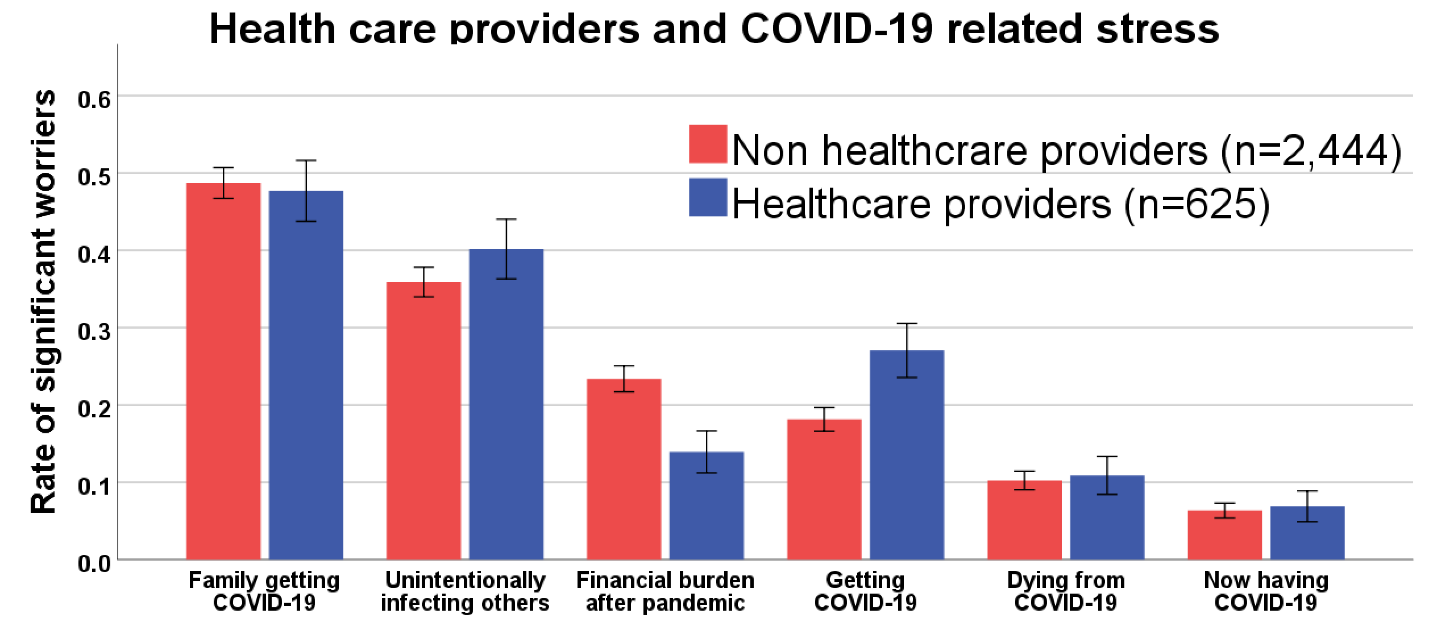


Caption: Y- axis represents the rate of responders endorsing significant worry (a lot/a great deal, items 4/5 on a 5 option Likert scale). Error bars represent 95% confidence intervals.

**Supplementary eFigure 2-** Anxiety and depression comparing healthcare providers to non-healthcare providers.


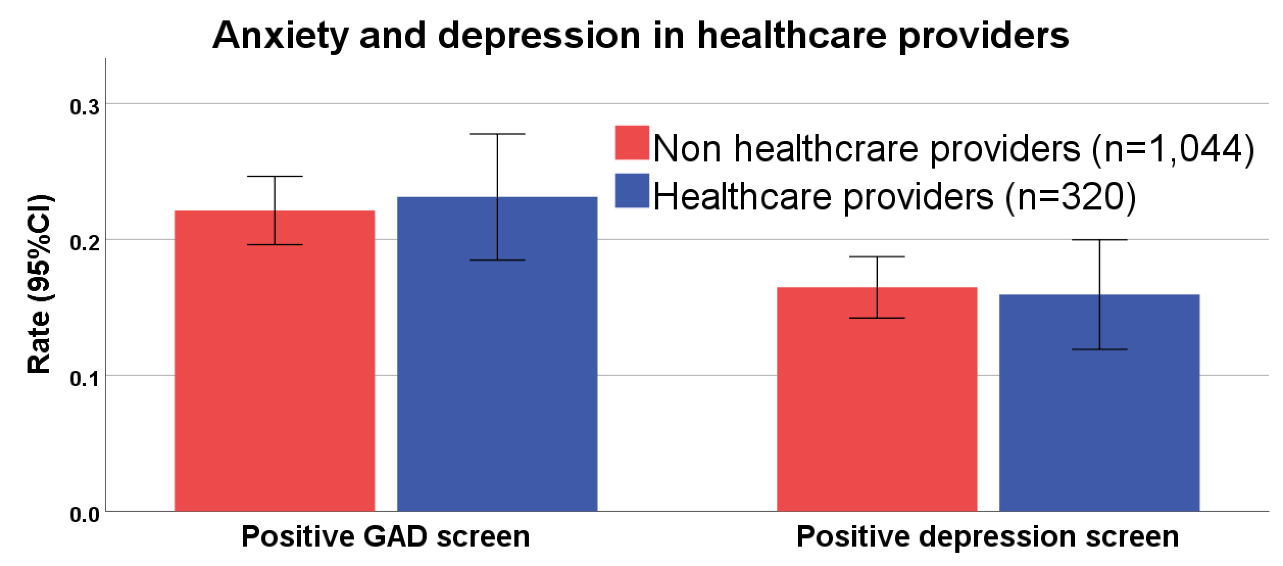


Caption: A positive GAD screen was considered for in GAD7 score >10. Positive depression screen was considered for PHQ2 score>2. GAD= generalized anxiety disorder.

**Supplementary eFigure 3-** Resilience profile and COVID-19 related worries comparing healthcare providers to non-healthcare providers.


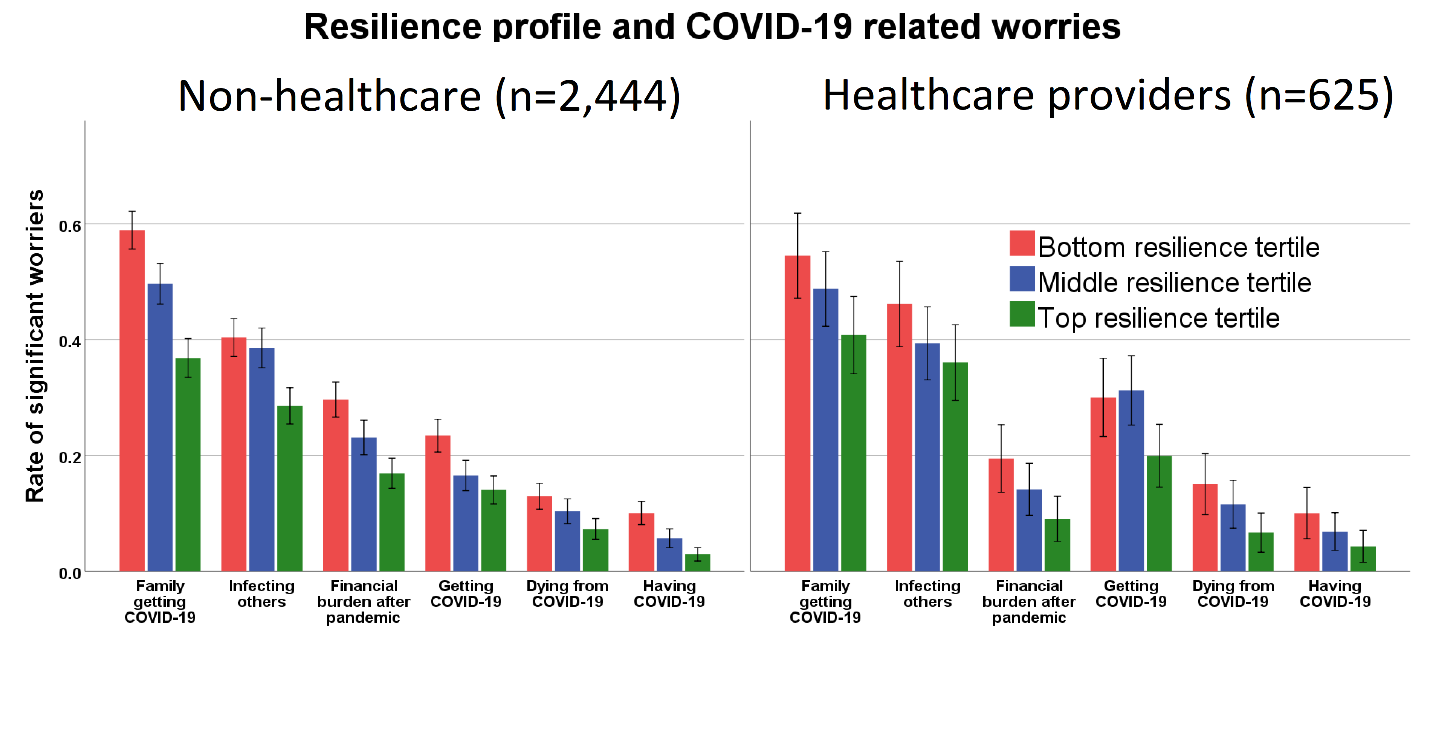
Caption: Y- axis represents the rate of responders endorsing significant worry (a lot/a great deal, items 4/5 on a 5 option Likert scale). Error bars represent 95% confidence intervals.

**Supplementary eFigure 4-** Resilience profile, anxiety and depression comparing healthcare providers to non-healthcare providers.


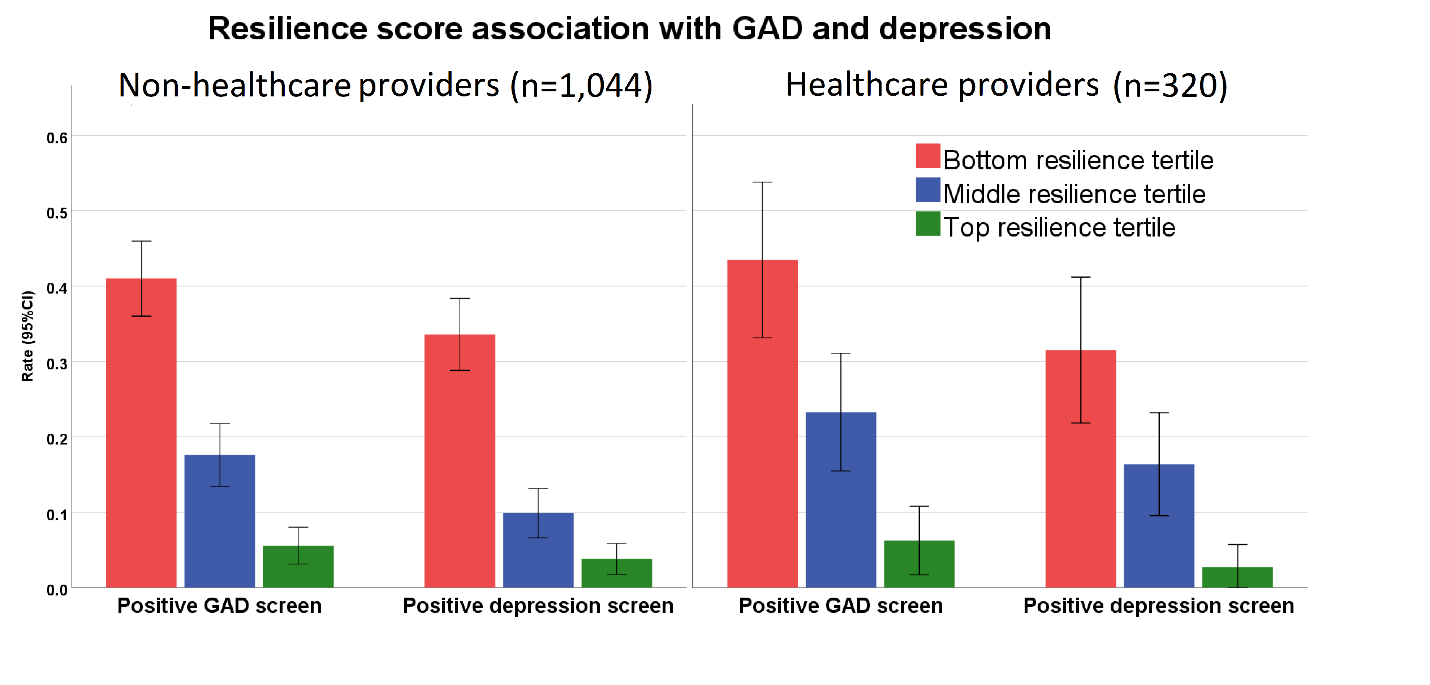


Caption: A positive GAD screen was considered for in GAD7 score >10. Positive depression screen was considered for PHQ2 score>2. GAD= generalized anxiety disorder.

**Supplementary eTable 1**- Resilience survey items.

|  | Scale Range | Mean | SD |
| --- | --- | --- | --- |
| When I'm in a difficult situation, I can usually find my way out of it. | 1-7 | 5.91 | 0.94 |
| I am determined. | 1-7 | 5.91 | 1.09 |
| My belief in myself gets me through hard times. | 1-7 | 5.53 | 1.29 |
| When I'm upset, I have difficulty focusing on other things^#^ | 1-5 | 3.27 | 1.03 |
| When I'm upset, I have difficulty concentrating^#^ | 1-5 | 3.27 | 1.04 |
| When I'm upset, I believe that I will end up feeling very depressed^#^ | 1-5 | 4.11 | 1.05 |
| when I'm upset, I have difficulty getting work done^#^ | 1-5 | 3.62 | 1.06 |
| when I'm upset, I have difficulty controlling my behaviors^#^ | 1-5 | 4.13 | 0.94 |
| How much does [Parent/Significant Other/Best Friend] treat you like you're admired and respected? | 1-5 | 3.89 | 1.01 |
| How sure are you that this [Parent/Significant Other/Best Friend] relationship will last no matter what? | 1-5 | 4.13 | 0.99 |
| How sure are you that this [Sibling/Best Friend] relationship will last no matter what? | 1-5 | 4.11 | 1.08 |
| How much does [Sibling/Best Friend] really care about you? | 1-5 | 3.95 | 1.02 |
| How much do you and [Parent/Significant Other/Best Friend] get annoyed with each other's behavior?^#^ | 1-5 | 4.00 | 0.78 |
| How much do you and [Parent/Significant Other/Best Friend] disagree and quarrel?^#^ | 1-5 | 4.26 | 0.71 |
| How much do you and [Parent/Significant Other/Best Friend] hassle or nag one another?^#^ | 1-5 | 4.25 | 0.78 |
| How much do you and [Parent/Significant Other/Best Friend] get on each other's nerves?*^#^* | 1-5 | 4.33 | 0.73 |
| How much do you and [the person with whom you have the second closest relationship] get on each other's nerves?^#^ | 1-5 | 4.29 | 0.73 |
| My neighborhood is safe from crime. | 1-5 | 4.06 | 0.96 |
| Violence is not a problem in my neighborhood. | 1-5 | 4.26 | 0.92 |
| I feel safe walking in my neighborhood, day or night. | 1-5 | 4.29 | 0.90 |
| People in my neighborhood can be trusted. | 1-5 | 4.05 | 0.82 |

^#^These items were reverse codes so that higher score correlates with resilience.

**Supplementary eTable 2-** Associations of different COVID-19 related worries with anxiety and depression screening.

|  |  | OR | Low 95%CI | High 95%CI | P-value |
| --- | --- | --- | --- | --- | --- |
| GAD positive screen | COVID-19 worries about self^#^ | 2.34 | 1.89 | 2.89 | <.001 |
|  | COVID-19 worries about others^&^ | 2.36 | 1.88 | 2.95 | <.001 |
|  | Financial worries d/t COVID-19 | 1.87 | 1.57 | 2.23 | <.001 |
| PHQ positive screen | COVID-19 worries about self | 1.56 | 1.26 | 1.93 | <.001 |
|  | COVID-19 worries about others | 1.71 | 1.36 | 2.15 | <.001 |
|  | Financial worries d/t COVID-19 | 1.8 | 1.5 | 2.15 | <.001 |

Values are derived from binary logistic regressions with all three worry types as independent variables (the three worry types were regressed out of each other to account for co-linearity among worries) and positive GAD screen (GAD7 score>10) or PHQ screen (PHQ2>2) as the dependent variable. Models co-varied for age, gender, race, marital status, income, education, occupation, number of people in household, country and date taking the survey.

^#^Worries about self include: Contracting COVID-19; Dying from COVID-19; Now having COVID-19.

^&^Worries about others include: Family contracting COVID-19; Unintentionally infecting others with COVID-19;

Abbreviations: PHQ=Patient Health Questionnaire-2; GAD=Generalized Anxiety Disorder Questionnaire-7.

**Supplementary eTable 3-** Demographic comparison between US and Israel participants.

|  | US (n=1607) | | Israel (n=1197) | |  |  |
| --- | --- | --- | --- | --- | --- | --- |
| Demographics | Mean | SD | Mean | SD | t | P-value |
| Age, years | 38.74 | 12.14 | 38.94 | 11.43 | 0.443 | 0.658 |
| Number in household | 2.91 | 1.46 | 3.61 | 1.79 | 10.98 | <.001 |
|  | n | % | n | % | Chi-square | P-value |
| Female gender | 1344 | 83.7 | 470 | 39.3 | 592 | <.001 |
| Married | 1089 | 67.8 | 906 | 75.7 | 21 | <.001 |
| Education Master/Doctoral | 940 | 58.6 | 554 | 46.6 | 144 | <.001 |
| Income > $100k | 889 | 59.1 | 339 | 31.1 | 398 | <.001 |
| Healthcare providers | 459 | 28.6 | 130 | 10.9 | 129 | <.001 |
| COVID exposures | n | % | n | % | Chi-square | P-value |
| Tested | 78 | 4.9 | 66 | 5.6 | 8.6 | 0.124 |
| Know someone tested+ | 742 | 46.2 | 445 | 37.2 | 34.1 | <.001 |
| Know someone died | 114 | 7.1 | 61 | 5.1 | 10.7 | 0.013 |
| Survey factors | n | % | n | % | Chi-square | P-value |
| Took PHQ/GAD | 831 | 51.7 | 424 | 35.4 | 73.6 | <.001 |
| Positive GAD screen | 245 | 29.5 | 35 | 8.3 | 73 | <.001 |
| Positive PHQ screen | 143 | 17.2 | 54 | 12.7 | 4.2 | 0.039 |

Abbreviations: PHQ=Patient Health Questionnaire-2; GAD=Generalized Anxiety Disorder Questionnaire-7.
